# Repair of distal thumb degloving injury using combination of reverse dorsoradial flap of the thumb and middle finger proper digital arterial island flap

**DOI:** 10.1186/s13018-020-01940-y

**Published:** 2020-09-16

**Authors:** Ruizheng Hao, Bin Wang, Hui Wang, Huanyou Yang, Yongxin Huo

**Affiliations:** Department of Hand Surgery, The Second Hospital of Tangshan, No.21 Jianshe North Road, Lubei District, Tangshan, 063000 Hebei Province People’s Republic of China

**Keywords:** Distal thumb degloving injury, Reverse dorsoradial flap of the thumb, Middle finger proper digital arterial island flap

## Abstract

**Background:**

To examine the efficacy of combination of the reverse dorsoradial flap of the thumb and middle finger proper digital arterial island flap for repair of distal thumb degloving injury.

**Methods:**

Twelve patients with mechanical distal thumb degloving injuries were treated between February 2017 and August 2019. A combination of the reverse dorsoradial flap of the thumb and the middle finger proper digital arterial island flap were used. Semmes–Weinstein (SW) monofilament and static two-point discrimination (S-2PD) tests, active range-of-motion (ROM) of the joints, cold intolerance, visual analog scale (VAS) score patient complications, and patient satisfaction were sequentially evaluated.

**Results:**

Two cases with postoperative flap blisters were treated at time of dressing changes up to successful scab formation. One case with postoperative arterial crisis of finger arterial dorsal branch vessel was successfully released in the pedicle. Ten cases healed by first intention and 2 cases by secondary intention. Twelve patients received follow-up examinations between 3 and 20 months (average 13 months) post-treatment, and all exhibited full, soft flaps with no fingertip pain.

**Conclusion:**

The combined use of the reverse dorsoradial flap of the thumb and the middle finger proper digital arterial island flap is a practical and effective approach to surgical repair of distal thumb degloving injuries.

## Background

Distal degloving injuries are common, but reconstruction of the thumb remains a challenge for surgeons [1]. Several procedures, including osteoplastic reconstruction with transferred pedicles of abdominal or forearm tissue and free vascularized transfer of the first or the second finger, have been tried in efforts to improve functional and aesthetic outcomes of thumb reconstruction. While treatment involving thumb shortening and suturing is straightforward, postoperatively the resulting abnormal thumb length has serious functional consequences [2]. A ventral skin tube can restore thumb length [3, 4], but the procedure requires a high degree of technical expertise and is associated with multiple drawbacks including bloated appearance, poor sensory outcomes, difficult thumb and nail free grafting, and high risk of surgical injuries [5–9].

Currently, the reverse dorsoradial flap of the thumb or the middle finger proper digital arterial island flap is widely used to repair thumb tip defects [10, 11]. However, due to the limited area that can be repaired using a single flap, neither approach on its own is suitable for treating thumb degloving injuries involving large defective areas. Therefore in this study, we aim to assess the combined use of the reverse dorsoradial flap of the thumb with middle finger proper digital arterial island flap to repair twelve cases of distal thumb degloving injuries.

## Methods

### Patients

A total of 12 patients with mechanical thumb degloving injuries treated in our hospital from February 2017 and August 2019 were retrospectively included. There were 7 men and 5 women. Patients ranged in age from 19 to 62 years (average age 32 years). The time from injury to surgery was from 1 to 6 h (average 3 h). The defects involved soft tissue detachment of 1/2 distal thumb (*n* = 3), 2/3 of distal thumb (*n* = 7) and 3/4 distal thumb (*n* = 2). The defect size of thumb degloving injury was in length from 1.0 to 3.3 cm and in width from 2.5 to 3.5 cm on the palmar side, and in length from 1.5 to 3.2 cm and in width from 2.5 to 3.5 cm on the dorsal side. The injured thumb was involved mild to severe contamination and exposure of tendon and phalange. There was no rupture of flexor and extensor tendons. There were 4 cases with missing nail bed and 8 cases with damaged nail bed. Two cases were associated with distal fractures. Patient demographics are listed in Table 1. Signed informed consent was obtained from each patient. The study was approved by the Ethics Committee of Tangshan Second Hospital (TSEY-LL-2020016).

**Table 1:**
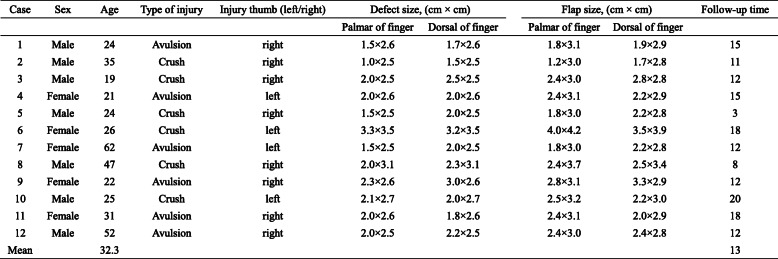
Demographic and surgical details of the patients

Inclusion criteria were patients with distal skin and soft tissue defects at the thumb interphalangeal joint, with or without distal phalanx fractures. Exclusion criteria were patients with (1) bone lesions, (2) a history of rheumatoid arthropathy, (3) diabetes and (4) tuberculosis.

### Surgical methods

The surgery was all performed by the same senior surgeon. Patients were administered brachial plexus block anesthesia. A tourniquet was applied to the injury-bearing arm. Wound debridement was performed to remove necrotic and contaminated tissue, nail matrix and residual nail bed. One- to 1.2-mm Kirschner wire was used for fixation in cases of distal fractures.

**Fig. 1:**
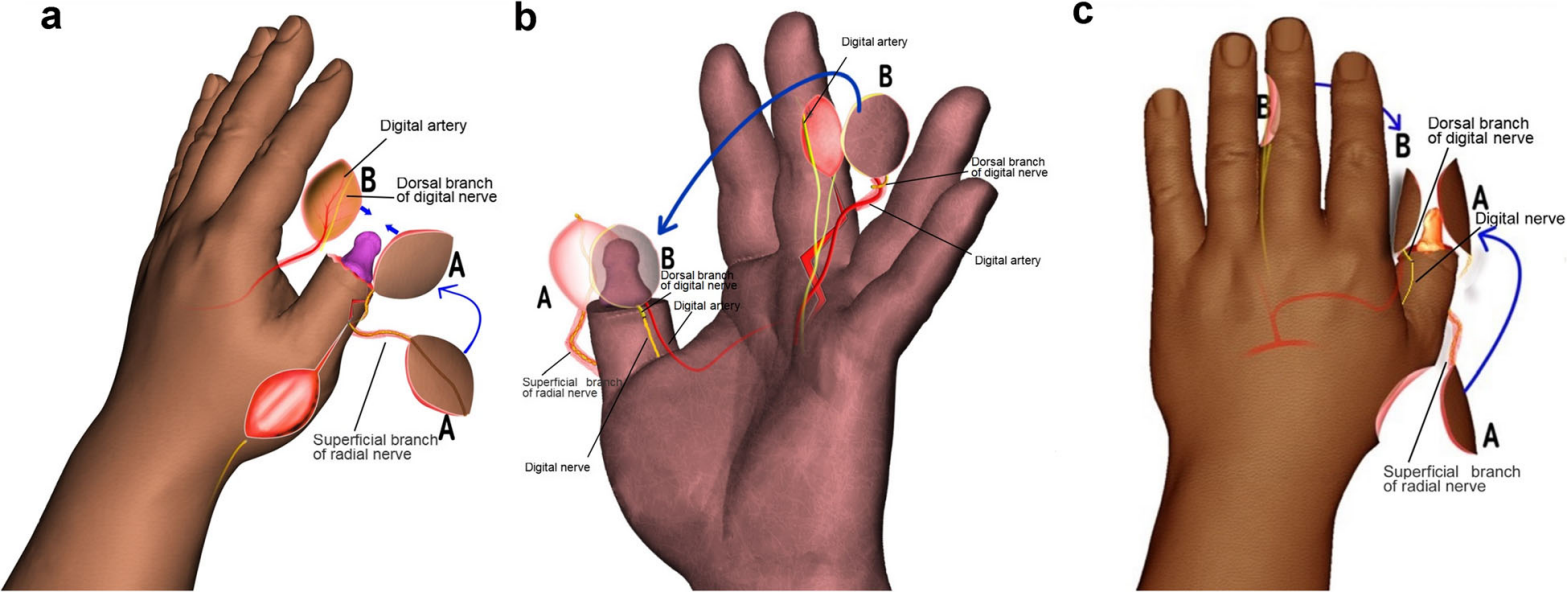
**a** The reverse dorsoradial flap of the thumb was designed on the radial dorsal area of the thumb according to the size of the skin defect on the dorsal side of the thumb. The rotation point is located on the radial side of the thumb interphalangeal joint. The axis aligned the radial side of the carpal joint with the radial side of the interphalangeal joint. The flap and pedicle included the superficial branch of radial nerve of dorsal thumb. **b** The middle finger proper digital arterial island flap was then designed according to the injury size of the palmar thumb. The flap contains dorsal branches of the proper digital nerve. The pedicle of the flap was designed as a narrow shape encompassing the proper palmar digital artery of the ulnar side of the middle finger. The radial proper digital artery of the ring finger was cut off and ligated at the beginning. **c** Under the microscope, the dorsal branch of the digital proper nerve was anastomosed with the ulnar proper nerve of the thumb. The reverse dorsoradial flap of the thumb and the middle finger proper digital arterial island flap were used to repair the degloving injury of the distal thumb

The reverse dorsoradial flap of the thumb was designed on the radial dorsal area of the thumb according to the size of the skin defect on the dorsal side of the thumb. The area of the flap was 10% larger than the dorsal surface of the thumb. The rotation point is located on the radial side of the thumb interphalangeal joint (Fig. 1a). The axis aligned the radial side of the carpal joint with the radial side of the interphalangeal joint. The flap and pedicle included the superficial branch of radial nerve of dorsal thumb. A 0.5- to 0.6-cm fascia pedicle was retained around the vascular pedicle to increase arterial blood supply and flap venous return. The pedicle was 0.8 to 1.0 cm in length. The proximal part of the flap could extend to the proximal part of the first metacarpal bone, and the distal part could reach the middle section of the proximal phalanx of the thumb. The width of the pedicle should not exceed the dorsal digit *d* of proximal thumb. The flap pedicle length is 0.8 to 1.0 cm, the proximal end of the flap can reach the proximal end of the first metacarpal, and the distal end can reach the middle section of the proximal phalanx of the thumb. Care was taken that the width of the pedicle did not exceed the dorsal median line of proximal thumb.

The middle finger anterograde ulnar proper digital arterial island flap was then designed according to the injury size of the palmar thumb (Fig. 1b). The dorsal thumb flap was cut 10% larger, and the palmar flap area was approximately 12% larger than the palmar wound. The healing wound should be on the dorsal side to avoid fingertip pain or bone exposure from scar stimulation on the bone terminus. The pedicle of the flap was designed as a narrow shape encompassing the proper digital artery and the dorsal branch of the proper digital nerve of the ulnar side of the middle finger. The radial proper digital artery of the ring finger was cut off and ligated at the beginning. The rotation point was located at the site of the superficial palmar arch of the common artery. The axis was the direction of the proper palmar digital artery of the ulnar side of the middle finger. Hemostatic forceps were then used to tease the tissues apart to make a subcutaneous passage between the palm and the thumb. A lead-wire pre-attached to the flap was guided through the passage and pulled to the palm side of the thumb, covering the palmar thumb. Under the microscope, the dorsal branch of the digital proper nerve was anastomosed with the ulnar proper nerve of the thumb (Fig. 1c). The reverse dorsoradial flap of the thumb and the middle finger proper digital arterial island flap were used to repair the degloving injury of the distal thumb. Forearm full thickness skin equivalent in area to the flap donor area was excised and used for grafting to repairing the donor sites. Anti-inflammation, detumescence treatments were administered following surgery. The sutures were removed after 14 days. Active functional exercises were then initiated.

### Evaluation of outcomes

Sensibilities of the flap and the donor site were measured using Semmes–Weinstein (SW) monofilament and static two-point discrimination (S-2PD) tests. The range of motion (ROM) of the thumb was measured with a standard hand goniometer, and the degree of flexion of the metacarpophalangeal and interphalangeal joints of the thumb minus the degree of extension loss was compared with the other hand. Cold intolerance of the reconstructed finger was also measured using the self-administered Cold Intolerance Severity Score questionnaire (CISS) [12]. The scores are grouped into four degrees (mild, moderate, severe, and extremely severe) corresponding to four ranges (0–25, 26–50, 51–75 and 76–100), respectively. Pain was given subjectively by the patient using the visual analog scale (VAS). The VAS consists of a 10-cm line that was grouped into mild (0–3 cm), moderate (4–6 cm), and severe (7–10 cm). The appearance of the reconstructed finger and the donor site were assessed using the Michigan Hand Outcomes Questionnaire [13, 14]. All tests were performed by the same senior surgeon.

## Results

Two cases with postoperative flap blisters were treated at time of dressing changes up to successful scab formation. One case with postoperative arterial crisis of finger arterial dorsal branch vessel was successfully treated symptomatically (i.e. suture removal, etc.). Ten cases healed by first intention and two cases by secondary intention. Twelve patients received follow-up examinations between 3 and 20 months (average 13 months) post-treatment, and all exhibited full, soft flaps with no fingertip pain.

As shown in Table 2, the mean S-2PD score of the reverse dorsoradial flap of the thumb was 6 to 11 mm (average 9 mm). The mean S-2PD score of the middle finger proper digital arterial island flap was 5 to 9 mm (average 7 mm). The mean SWM score of the reverse dorsoradial flap of the thumb was 2.83 to 4.61 mm (average 4.03 mm). The mean SWM score of the middle finger proper digital arterial island flap was 3.47 to 4.32 mm (average 3.85 mm). Based on the CISS score, 9 patients of reverse dorsoradial flap of the thumb reported no cold intolerance and 3 reported mild cold intolerance. Ten patients of the middle finger proper digital arterial island flap reported no cold intolerance and 2 reported mild cold intolerance. According to the VAS score, 9 patients had no pain, 2 reported mild pain and 1 has moderate pain on the middle finger proper digital arterial island flap. Ten patients had no pain, and 2 reported mild pain on the reverse dorsoradial flap of the thumb.

**Table 2:** Postoperative assessment of injured finger

	Palmar of finger				Dorsal of finger			
Case	S-2PD, mm	SWM	Cold Intolerance	Pain	S-2PD, mm	SWM	Cold Intolerance	Pain
1	7	3.65	0	0	9	4.61	0	0
2	7	4.12	20	1	6	4.28	20	0
3	9	4.32	0	0	11	3.76	0	0
4	7	3.65	0	0	10	4.32	0	0
5	9	3.75	0	0	10	2.83	10	2
6	6	4.24	0	2	8	4.28	0	0
7	7	3.65	0	0	9	3.96	0	2
8	9	4.15	10	0	11	4.28	0	0
9	9	3.85	0	0	8	3.59	0	0
10	5	3.51	0	0	10	3.61	0	4
11	8	3.47	0	0	7	4.28	20	0
12	9	3.84	0	0	9	4.56	0	0
Mean	7	3.85			9	4.03		

*S-2PD* static 2-point discrimination, *SWM* Semmes–Weinstein monofilament

The active ROM of metacarpophalangeal (MCP) and interphalangeal (IP) joints of the injured thumbs were satisfactory (Table 3). No statistical differences were observed in the ROM of MCP and IP joints compared with that of the contralateral side (pMCP = 0.185, pIP = 0.137). The quality of activity of the injured thumbs showed no abnormality.

**Table 3:** ROM assessment of the thumbs

	ROM (degree)			
CASE	MCPI	MCPC	IPI	IPC
1	80	80	30	30
2	40	45	75	80
3	60	57	70	78
4	60	60	65	70
5	65	70	65	65
6	65	65	72	70
7	60	55	60	60
8	65	62	68	75
9	54	65	72	66
10	75	80	65	65
11	70	80	55	75
12	80	80	70	70
Mean	64.50	66.58	63.92	67.00
*t*	1.431 0.185		1.602	
*P*			0.137	

*ROM* range of motion, *MCPI* metacarpophalangeal joint of injury side, *MCPC* metacarpophalangeal joint of contralateral side, *IPI* interphalangeal joint of injury side, *IPC* interphalangeal joints of contralateral side

According to the Michigan Hand Outcomes questionnaire, 9 patients were strongly satisfied (score of 5) and 3 patients were satisfied (score of 4) with the appearance, whereas 10 patients were strongly satisfied (score of 5) and 2 patients were satisfied (score 4) with the function of the reconstructed thumb.

## Case reports

**Fig. 2:**
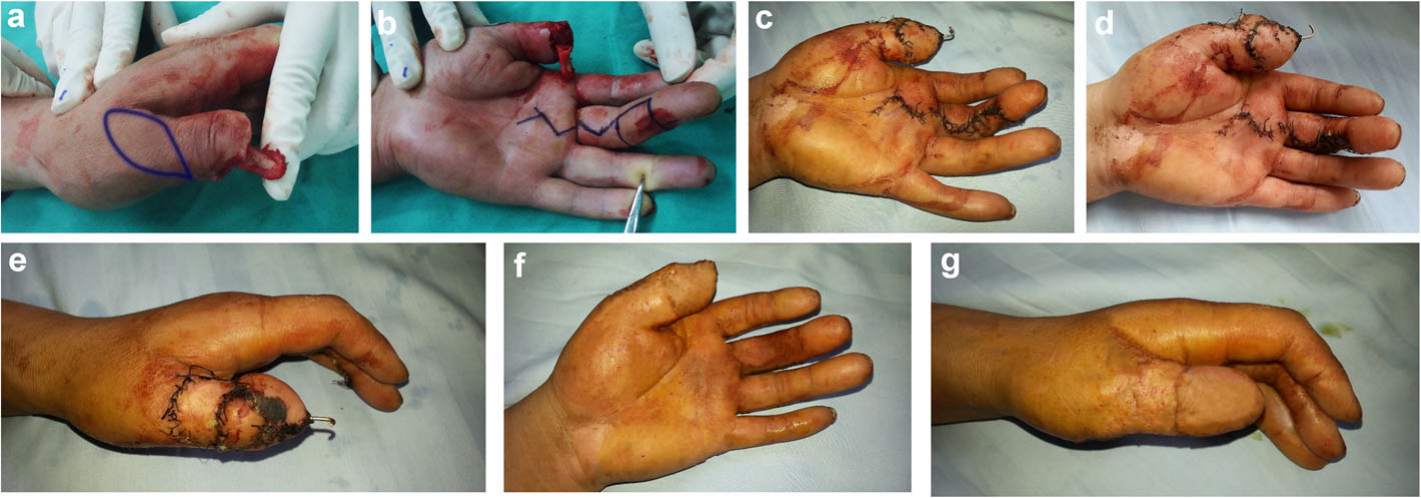
Flap of case 4. **a**, **b** The size of the defect were 2.0 cm × 2.6 cm on the palm side and 2.0 cm × 2.6 cm on the dorsal side. **c**–**e** Skin flap cutting and suturing. **f**, **g** The appearance of the flaps 15 months later

Case 4: A 21-year-old female with left thumb distal tissue avulsion injury. The size of the defect was 2.0 cm × 2.6 cm on the palm side and 2.0 cm × 2.6 cm on the dorsal side (Fig. 2a, b). A 2.4 cm × 3.1 cm size reverse dorsoradial flap of the thumb and a 2.2 cm × 2.9 cm size middle finger proper digital arterial island flap were raised to reconstruct the defect (Fig. 2c–e). At the 15-month follow-up evaluation, good finger function and satisfactory appearance were obtained (Fig. 2f, g). The mean S-2PD score of the reverse dorsoradial flap of the thumb and the middle finger proper digital arterial island flap was 7 mm and 10 mm. The ROM of MCP and IP joints were 60 and 65°, respectively.

**Fig. 3:**
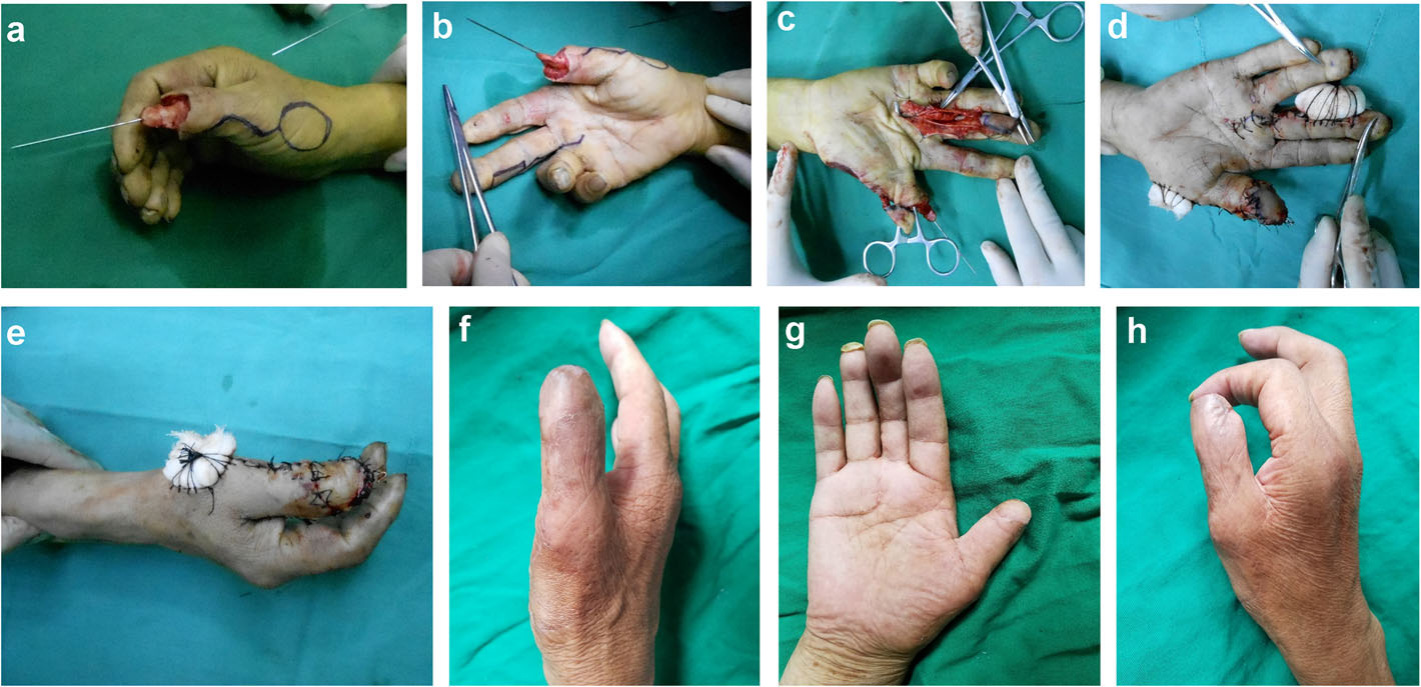
Flap of case 11. **a**, **b** The size of the defect were 2.0 cm × 2.6 cm on the palm side and 1.8 cm × 2.6 cm on the dorsal side. **c** The flaps were cut. **d**, **e** After skin flap suturing. **f**–**h** The appearance of the flaps 18 months later

*Case 11***:** A 31-year-old female with right thumb distal tissue avulsion injury. The size of the defect was 2.0 cm × 2.6 cm on the palm side and 1.8 cm × 2.6 cm on the dorsal side (Fig. 3a, b). A 2.4 cm × 3.1 cm size reverse dorsoradial flap of the thumb and a 2.0 cm × 2.9 cm size middle finger proper digital arterial island flap were raised to reconstruct the defect (Fig. 3c–e). At the 18-month follow-up evaluation, good finger function and satisfactory appearance were obtained (Fig. 3f–h). The mean S-2PD score of the reverse dorsoradial flap of the thumb and the middle finger proper digital arterial island flap was 5 mm and 9 mm. The ROM of MCP and IP joints were 70 and 80°, respectively.

## Discussion

Restoring the original length is important for functional recovery in repairing a traumatic thumb injury [15]. Local transposition flaps, advancing flaps and homo-finger neurovascular island flaps are common methods for repairing soft tissue defects at the fingertips, but these methods are only applicable to small to medium-sized defects (length < 2 cm) [16, 17]. Free toe flap plus hand island flap technique provides a good-looking thumb with a good sensitivity recovery, but free flap surgery is cumbersome, difficult to operate and complicated to operate and requires a high degree of skills [18]. In addition, for the middle finger island flaps [19], the middle and ring finger island flaps [20] and the cutaneous neurotrophic flap plus the middle (ring) island flap [21], 2 blood vessels of the 2 flaps need to be separated during surgery.

The reverse dorsoradial flap of the thumb and the middle finger proper digital arterial island flap are used to repair the skin and soft tissue defects on the dorsal or palm side of the thumb [22–24]. However, due to the limited cutting range of one of the two flaps, it is impossible to effectively repair the dorsal and palmar defects caused by thumb degloving injury. At this time, combining the two flaps will be a good innovative choice. In this study, the reverse dorsoradial flap of the thumb was used to repair the dorsal soft tissue defect of the thumb. The middle finger proper digital arterial island flap was used to repair the soft tissue defect of thumb palmar side and reconstruct the skin sensation of thumb palmar side. The combination of the two flaps provides a new method for repairing the soft tissue defect of distal thumb.

In our study, reconstructed finger exhibited excellent skin condition and highly satisfactory aesthetic appearance and shape according to the Michigan Hand Outcomes questionnaire. The double-leaf flaps repaired large areas of sleeve-like skin injury and restored maximum thumb length using relatively simple operation with the lateral thumb flap beside the same finger and reasonably close flap pedicle rotation and transfer distance. Large injury can be repaired in a single operation with short treatment cycle, simple post-operative care and early onset of rehabilitation training to facilitate recovery of hand function. There is no difference between the active ROM of MCP and IP joints with the contralateral side.

The indications and precautions of our surgical method are as follows. (1) When designing the flap, care should be taken that the length of the middle finger proper digital arterial island flap is greater than the reverse dorsoradial flap of the thumb. In addition, the healing wound should be on the dorsal side to avoid fingertip pain or bone exposure from scar stimulation on the bone terminus. (2) A 0.5- to 0.6-cm fascia pedicle should be retained around the vascular pedicle to increase arterial blood supply and flap venous return [25]. (3) To treat flap venous obstacles, suture removal and tension reduction should be performed 1 to 3 days after surgery. Moreover, the open tunnel should be wide enough to pass through the flap pedicle. (4) Reconstruction of the finger intrinsic nerve sensory components of the middle finger proper digital arterial island flap should be performed so as to restore finger pulp sensation [26–28]. The nerve separation and anastomosis procedures must be performed using a microscope. (5) Residual damaged nail beds require removal during surgery to avoid inlay formation. (6) Skin graft packing pressure must be appropriate but not excessive, to avoid too much pressure being transmitted to the pedicle and thereby affecting blood supply to the flap. (7) Tension must be avoided when suturing flaps. Pressure must not be applied when covering with gauze and dressing pedicles.

Several considerations should be taken when performing surgery: (1) The flap should be of sufficient width and length so that the two flaps can heal without tension. (2) The pedicle passage of these two flaps should be opened as wide as possible to avoid being oppressed when the flap flips is turned over. (3) The anterograde island flap on the flank of the middle finger should allow for a loose subcutaneous passage to avoid pressure on the pedicle affecting blood circulation. (4) A postoperative drainage strip should be placed to prevent hematoma formation affecting flap blood supply. (5) Loop-like small vessels should be abundant around the superficial branch of the covered nerve in the flaps, in order to insure an adequate blood supply to the nerve. (6) Care should be taken to protect the tendon superficial fascia in order to insure survival of the skin graft and avoid tendon adhesion. (7) The axial length of the middle finger proper digital arterial island flap should exceed the injury length in the pulp, and the suture mouth should be located on the dorsal side of the distal phalange during imbrication suturing, in order to avoid the later appearance of damaged skin at the distal end of the phalange.

There were some limitations of this study. For the method, both flap donor sites are located on the hand and the incision is large; the flap donor site requires repair with skin grafts. The onychostroma of the thumb is completely removed, and there is no nail plate growth after surgery, compromising hand integrity. In addition, large randomized controlled study should be carried out in the future to further explore long-term efficacy.

## Conclusion

The reverse dorsoradial flap of the thumb combined with the middle finger proper digital arterial island flap for the treatment of the degloving injury of the distal thumb can repair the skin defect at one time, reconstruct the good feeling of the finger pulp and obtain satisfactory effect.

## Abbreviations

SW: Semmes–Weinstein
S-2PD: Static two-point discrimination
ROM: Range-of-motion
VAS: Visual analog scale
CISS: Cold Intolerance Severity Score questionnaire

## References

[1] Feng SM, Gu JX, Liu HJ, Zhang NC, Pan JB, Tian H (2013). Treatment of distal fingertip degloving injuries using a cross-finger flap based on the dorsal branch of the proper digital artery at the middle phalanx. J Reconstr Microsurg.

[2] Venkataswami R, Subramanian N (1980). Oblique triangular flap: a new method of repair for oblique amputations of the fingertip and thumb. Plast Reconstr Surg.

[3] Ju J, Li J, Hou R (2015). Microsurgery in 46 cases with total hand degloving injury. Asian J Surg.

[4] Noaman HH (2012). Salvage of complete degloved digits with reversed vascularized pedicled forearm flap: a new technique. J Hand Surg Am.

[5] Ray EC, Sherman R, Stevanovic M (2009). Immediate reconstruction of a nonreplantable thumb amputation by great toe transfer. Plast Reconstr Surg.

[6] Ornelli M, Ruocco G, Kaciulyte J, Lazzaro L, Felici N (2019). Immediate vs. delayed toe-to-thumb transfer: is the infection rate greater?. Handchir Mikrochir Plast Chir.

[7] Liu C, Liu L, Liu G, Tian S, Bai J, Yu K (2019). Tian D repair of thumb defect by using the toenail flap: biomechanical analysis of donor foot-a retrospective cohort study. J Orthop Surg Res.

[8] Yang K, Zhao Z, Pan Y, Song F, Deng J, Zhu J Resorption of iliac bone grafts following wrap-around flap for thumb reconstruction: a follow-up study. J Hand Surg Am 2020;45:64.e1-.e8. 10.1016/j.jhsa.2019.03.010 31076269

[9] Xuefeng Z, Jian G, Jiayue D, Chuchen Z, Shenglin W (2020). Xiao'en Y clinical effect of thumb finger reconstruction using dorsal foot flap transplant for treating thumb defects. Med Hypotheses.

[10] Büchler UFH (1988). The dorsal middle phalangeal finger flap. Handchir Mikrochir Plast Chir.

[11] Adani R, Marcoccio I, Tarallo L, Fregni U (2005). The reverse heterodigital neurovascular island flap for digital pulp reconstruction. Tech Hand Up Extrem Surg.

[12] Irwin MS, Gilbert SE, Terenghi G, Smith RW, Green CJ Cold intolerance following peripheral nerve injury. Natural history and factors predicting severity of symptoms. J Hand Surg Br 1997;22:308-316. 10.1016/s0266-7681(97)80392-0 9222907

[13] Chung KC, Pillsbury MS, Walters MR, Hayward RA (1998). Reliability and validity testing of the Michigan hand outcomes questionnaire. J Hand Surg Am.

[14] Chung KC, Hamill JB, Walters MR, Hayward RA (1999). The Michigan hand outcomes questionnaire (MHQ): assessment of responsiveness to clinical change. Ann Plast Surg.

[15] Cao XCJ (2002). Double mini-flaps from fingers for reconstruction of distal portion of thumb. Hand Surg.

[16] Chen C, Tang P, Zhang X A comparison of the dorsal digital island flap with the dorsal branch of the digital nerve versus the dorsal digital nerve for fingertip and finger pulp reconstruction. Plast Reconstr Surg 2014;133:165e-73e. 10.1097/PRS.0000000000000057 24469187

[17] Brambullo T, Dalla Venezia E, Vindigni V, Bassetto F (2018). Extended Hueston flap: new solution for primary closure. J Hand Microsurg.

[18] Wang B, Jia S, Lu A, Hao R, Huo Y, Fei X (2016). Pang H [reconstruction of degloved thumbs with free second toe dorsal flap combined with middle or ring finger island flap]. Zhongguo Xiu Fu Chong Jian Wai Ke Za Zhi.

[19] Yao JM, Song JL, Xu JH (1996). The second web bilobed island flap for thumb reconstruction. Br J Plast Surg.

[20] Qi W, Chen KJ (2013). Use of twin dorsal middle phalangeal finger flaps for thumb or index finger reconstruction. J Hand Surg Eur Vol.

[21] Zunjiang Z, Zongsheng Y, Yong L, Yudong H (2013). Reconstruction of an electric burn in the ventral thumb using the middle ring digital artery island flap with nerve. Burns.

[22] Pagliei A, Rocchi L, Tulli A (2003). The dorsal flap of the first web. J Hand Surg Br.

[23] Hu D, Wei Z, Wang T, Hong X, Zheng H, Lin J (2020). Anatomical basis and clinical application of the dorsal perforator flap based on the palmar artery in the first web. Surg Radiol Anat.

[24] Zhang X, He Y, Shao X, Li Y, Wen S, Zhu H (2009). Second dorsal metacarpal artery flap from the dorsum of the middle finger for coverage of volar thumb defect. J Hand Surg Am.

[25] Bertelli JAKZ (1992). Neurocutaneous island flaps in the hand:anatomical basis and preliminary results. Br J Plast Surg.

[26] Wang H, Yang X, Chen C, Huo Y, Wang B, Wang W Modified heterodigital neurovascular island flap for sensory reconstruction of pulp or volar soft tissue defect of digits. J Hand Surg Am 2020;45:67.e1-.e8. 10.1016/j.jhsa.2019.04.014 31235214

[27] Toros T, Gurbuz Y, Kelesoglu B, Ozaksar K, Sugun TS (2018). Reconstruction of extensive pulp defects of the thumb with a radial-based pedicled flap from the index finger. J Hand Surg Eur Vol.

[28] Chen C, Tang P, Zhang L, Li X, Zheng Y (2013). Repair of multiple finger defects using the dorsal homodigital island flaps. Injury.

